# Dietary Diversity, Haemoglobin and Anaemia in Nepali Adolescent Girls: A Longitudinal Study

**DOI:** 10.1111/mcn.70090

**Published:** 2025-08-29

**Authors:** Anjana Rai, Kenda Cunningham, Darren Wraith, Ramesh P. Adhikari, Marguerite C. Sendall, Naomi Saville, Smita Nambiar

**Affiliations:** ^1^ School of Public Health and Social Work Queensland University of Technology Brisbane Queensland Australia; ^2^ Helen Keller International London UK; ^3^ Helen Keller International Kathmandu Nepal; ^4^ Department of Environmental and Public Health, College of Health Sciences Abu Dhabi University Abu Dhabi United Arab Emirates; ^5^ Institute for Global Health University College London London UK; ^6^ School of Nutrition and Exercise Sciences Queensland University of Technology Brisbane Queensland Australia

**Keywords:** adolescents, anaemia, diets, girls, haemoglobin, Nepal, South Asia

## Abstract

Adolescent girls and young women in Nepal are vulnerable to poor diets and anaemia, yet the extent of these risks remains overlooked. We assessed changes in dietary diversity, haemoglobin, and anaemia, and identified associated factors among adolescent girls and young women in Nepal. We analysed data from a longitudinal panel study including never‐married and not‐pregnant participants, enroled at 10–19 years in 2017 (*n* = 770) and followed up in 2018 (*n* = 682) and 2019 (*n* = 618). We used descriptive statistics and mixed‐effects regression analyses. The dietary diversity score was on average four out of 10 food groups, haemoglobin remained between 12.7 and 12.8 g/dL throughout the study period, and anaemia prevalence increased from 20.6% (2017) to 24.8% (2019). In adjusted models, we found positive associations between more schooling and dietary diversity and between access to improved toilet and haemoglobin. Living in the *terai* and hills, and disadvantaged caste/ethnicity were negatively associated with dietary diversity, and haemoglobin, while living in the *terai* and disadvantaged caste/ethnicity were negatively associated with anaemia. Food insecurity was negatively associated with dietary diversity only. Post‐menarche status was associated with lower haemoglobin and higher odds of anaemia. Adolescent nutrition should be prioritised within national health, education, and social protection frameworks. Multi‐sectoral interventions particularly in *terai* and hills, should focus on scaling up micronutrient supplementation, enhancing government‐led school meal programme to provide balanced, culturally appropriate meals (including vegetarian protein sources for lacto‐vegetarians), improving educational uptake, ensuring access to sanitation facilities, and delivering targeted, sustained interventions around menarche throughout adolescence.

## Summary

- Post‐menarche girls show lower haemoglobin and higher anaemia risk, indicating critical windows for school‐ and community‐based micronutrient supplementation and nutrition education around menarche.
- Access to improved sanitation also correlates with better haemoglobin status, highlighting the importance of multisectoral approaches including WASH interventions.
- Greater schooling and household food security are positively associated with dietary diversity, though programmes must also address social and cultural barriers to further education, and equitable food access.
- Adolescent girls in Nepal's *terai* and hills and disadvantaged caste/ethnicity groups have lower dietary diversity and lower haemoglobin, while those in the *terai* and disadvantaged groups have higher anaemia prevalence, underscoring urgent need for targeted nutrition interventions.
- Further mixed‐method research on caste/ethnicity and agroecological zone disparities is needed to design equitable, context‐specific nutrition policies and programmes.

## 1 Introduction

Adolescence is a period of rapid physiological changes, including growth spurts, and maturation of reproductive and organ systems, that substantially increase nutritional requirements (Das et al. 2017). Adolescent girls in particular, are at a higher risk of micronutrient deficiencies due to menstrual blood loss and hormonal changes (Das et al. 2017; Ty Beal et al. 2024). Meeting these increased nutritional needs requires sustained and sufficient intake of macro‐ and micronutrient‐rich foods (Ty Beal et al. 2024; Chaparro and Suchdev 2019). However, adolescents and young women in South Asia often have poor quality diets that fail to meet recommended nutrient intake (Keats et al. 2018), predisposing them to micronutrient deficiencies, low haemoglobin and anaemia (Chaparro and Suchdev 2019). These have long‐term consequences for physical growth, cognitive development, work and educational performance (Madjdian et al. 2018) and future maternal and child health outcomes (Christian and Smith 2018; Patton et al. 2016).

In Nepal, the burden of undernutrition among adolescent girls and young women remains alarmingly high (Ministry of Health Nepal, New ERA, and ICF 2023). Adolescents' and young women's diets (10–25 years) fail to meet recommended nutrient needs (Diamond‐Smith et al. 2020; Singh et al. 2019; Morrison et al. 2023). The Nepal Demographic and Health Surveys indicate a slow decline in anaemia among adolescent girls 15–19 years from 44% in 2006 to 39% in 2022 (Ministry of Health Nepal, New ERA, and ICF 2023), persistently remaining a moderate to severe public health problem (World Health Organisation 2025). Studies from South Asia suggests dietary inadequacies and anaemia result from multifaceted biological, socioeconomic and behavioural factors (Madjdian et al. 2018; Diamond‐Smith et al. 2020; Singh et al. 2019; Morrison et al. 2023; Wiafe et al. 2023; Rai et al. 2023; Ford et al. 2022; van Tuijl et al. 2020; Estecha Querol et al. 2022; Baxter et al. 2021, 2022), further compounded by inequities related to gender, poverty, caste/ethnicity and geography, and individual agency (Christian and Smith 2018; Diamond‐Smith et al. 2020; Morrison et al. 2023; Rai et al. 2023; Blum et al. 2019; Sharma and Smieliauskas 2022).

Despite growing research on adolescent nutrition (Madjdian et al. 2018), research and data gaps persist (Choedon et al. 2024; Norris et al. 2022). Data on dietary (Ty Beal et al. 2024; Christian and Smith 2018) and micronutrient status (Christian and Smith 2018; van Tuijl et al. 2020) of adolescents and young women are limited and many studies neglect the contextual and social influences on nutrition (Madjdian et al. 2018; Estecha Querol et al. 2022). A recent scoping review of 295 studies on nutritional status had only 16 studies with longitudinal or pre‐post survey designs (Choedon et al. 2024). The predominance of cross‐sectional designs (Madjdian et al. 2018; Christian and Smith 2018) and limited number of longitudinal studies from South Asia restricts our ability to capture within‐individual changes in diets and nutrition (Miller et al. 2025). Given the slow progress in reducing anaemia and the reliance on poor‐quality diets among adolescents and young women, longitudinal studies are urgently needed to inform the development of effective, and context‐specific nutrition interventions.

To address these gaps, this study examines dietary diversity, haemoglobin and anaemia among adolescent girls and young women aged 10–22 years in Nepal using 3 years of longitudinal data. By covering early adolescence (10–14 years), late adolescence (15–19 years) and young adulthood (20–24 years), this study provides insights into how diets and anaemia change over time and the associated factors. We aim to inform policies and programme to improve adolescent girls' diet and reduce the burden of anaemia in Nepal and similar settings.

## 2 Methods

### 2.1 Setting

*Suaahara* II was a multi‐sectoral nutrition programme implemented in 42 of Nepal's 77 districts across six provinces, funded by the United States Agency for International Development and implemented by Helen Keller International and partners from 2016 to 2022 (Cunningham et al. 2020). The programme aimed to reduce maternal and child malnutrition, focusing on households in the 1000‐day period between conception and 2 years after birth. Additionally, *Suaahara* II had a learning agenda on adolescent nutrition through which it piloted an integrated health and nutrition package through a few sessions with limited students 10–19 years in 84 schools of four districts (Bardiya, Dailekh, Nawalparasi East and Nawalparasi West) aimed at improving adolescents' (10–19 years) health, nutrition, and water, sanitation, and hygiene (WASH) knowledge and practices (Cunningham et al. 2020).

Related to the learning agenda, *Suaahara* II carried out an adolescent panel study. Adolescent girls and mothers 10–19 years in 2017, residing in the households selected for 2017 survey were eligible for inclusion. Exclusion criteria were girls 20 years or older in 2017 or not providing consent/assent. Participants were subsequently also interviewed in 2018, 2019 and 2022 (Cunningham et al. 2020). For this analysis, we used data from girls 10–19 years in 2017, who were never married and not pregnant in 2017 (*n* = 770), never‐married and not pregnant at follow‐up in 2018 (*n* = 682) and never‐married and not pregnant in 2019 (*n* = 618). These criteria were selected to minimise confounding by marriage or pregnancy. This resulted in *N* = 2070 measurements over three study periods, with the oldest participants aged 22 in 2019. The final sample size for this analysis was determined by the number of eligible and consenting participants, rather than a priori power calculation. All eligible participants were included to allow for robust longitudinal analysis of growth patterns over the study period.

### 2.2 Outcomes of Interest

Outcomes were dietary diversity, haemoglobin, and anaemia. Dietary diversity was measured using the Minimum Dietary Diversity for Women (MDD‐W) tool, a standard for measuring population‐level dietary diversity, originally developed for women 15–49 years, and a proxy of micronutrient adequacy (FAO 2016; Arimond et al. 2010). Given the lack of a widely validated metric for adolescents and young women, and the application of MDD‐W in adolescent populations (Baxter et al. 2022; Bhatia et al. 2023; Aurino 2017; Diamond‐Smith et al. 2022), we justify its use for our sample while acknowledging that further validation in this group is warranted. MDD‐W uses 24 h recall data for 10 food groups: grains, starchy roots/tubers; pulses; nuts and seeds; dairy; meat, poultry, and fish; eggs; dark green leafy vegetables; other vitamin A‐rich fruits and vegetables; other vegetables; and other fruits (FAO 2016). We computed dietary diversity as the mean dietary diversity score (DDS) across these groups (FAO 2016; Arimond et al. 2010).

In the panel study, haemoglobin was measured using HemoCue® Hb‐301 photometers on capillary blood. Altitude, latitude, and longitude were recorded using Garmin eTrex 30x device, and smoking status was collected through a questionnaire. Haemoglobin values were adjusted for both smoking and elevation, as per World Health Organisation (2011) recommendations, by subtracting the recommended values from each individual's observed haemoglobin concentration (World Health Organisation 2011). We then used the adjusted haemoglobin levels to categorise anaemia as mild, moderate and severe using recommended cut‐offs for children 5–11 years, 12–14 years and nonpregnant women ≥ 15 years (World Health Organisation 2011). For the primary analysis, mild, moderate and severe anaemia categories were combined to define overall anaemia (anaemia vs. no anaemia).

### 2.3 Potential Associated Factors

Individual factors explored and retained in the final model for dietary diversity were age, level of education, lacto‐vegetarian diet (consuming dairy but excluding meat, fish and eggs). Household factors included food insecurity measured using household food insecurity access scale (HFIAS) (Coates et al. 2007), and socioeconomic status (SES) (Lower, Middle and Higher). Social factors included caste/ethnicity (socially disadvantaged vs. Brahmin/Chhetri vs. Others). Environmental factors included agro‐ecological zone (mountain, hill, *terai* (plains)).

For haemoglobin and anaemia, potential factors explored and retained in the final models were age, menarche status, lacto‐vegetarian, access to improved toilet (defined as access to a flush or pour‐flush toilet to a piped water system, septic tank or pit latrine or ventilated improved pit latrine or pit latrine with a slab, or composting toilet) (Ministry of Health Nepal, New ERA, and ICF 2023), SES, caste/ethnicity and agroecological zone.

### 2.4 Statistical Analyses

We used mean (SD) or median (IQR), percentages and plots to describe participant characteristics. We used mixed effects regression models to account for the repeated measures, and to model within‐ and between‐individual variations over time. The random intercept and slope were individual ID and age, respectively. The regression models included an unstructured covariance matrix to allow for unrestricted correlations between the random effects and included maximum likelihood estimation to estimate model parameters.

Bivariate linear mixed‐effects regression analyses were performed between all potential associated factors and DDS and haemoglobin. Variables with *p* < 0.2 in the bivariate results were considered for inclusion in the multivariable models. Starting with the full multivariable model, we conducted a stepwise removal of variables with largest *p*‐values. We used variance inflation factor (VIF) to check for multicollinearity between potential associated factors and checked for confounding at each step of variable removal by assessing changes in regression estimates by > 10%. The choice of final models were guided by clinical, contextual significance, and by Akaike Information Criterion and Bayesian Information Criterion. For anaemia, we replicated the final haemoglobin model and used logistic mixed effects regression analysis.

We predicted residuals and fitted values for final DDS and haemoglobin models to assess linear assumptions. While the violation of graphical linearity assumptions was not distinct for DDS and haemoglobin, as a sensitivity analysis approach we used bootstrapping (which implies fewer parametric assumptions) to account for potential uncertainty in the model and to estimate robust standard errors. Statistical significance was considered at *p* < 0.05% and 95% confidence intervals (CIs) were reported to provide a more comprehensive interpretation of results (Sedgwick et al. 2022). All analyses were conducted in Stata SE 17.0.

### 2.5 Ethics Statement

The Queensland University of Technology Human Research Ethics Committee provided waiver of ethical approval for this study as secondary datasets were used. Ethical approvals for the adolescent panel study from 2017 to 2019 were granted by the Nepal Health Research Council Approval reference nos. 1620, 2831, 3216).

## 3 Results

Table 1 shows participants' characteristics over 3 years. Median age of adolescent girls was 13.6 years, 14.3 years and 15.1 years in 2017, 2018 and 2019, respectively. Participants had a median education of Grade six, seven and eight over 3 years. More than half (51.9%) had reached menarche in 2017 which increased to 65.2% in 2018 and 78.3% in 2019. A small percentage of 4.8% (2017), 5.3% (2018) and 5.7% (2019) were lacto‐vegetarian over the study period. IFA supplementation was low but improved over time from 2% in 2017 to 16.9% in 2018 and 10.4% in 2019. Deworming (data available in 2018–2019 only) was higher in 2018 (31%) than in 2019 (21.4%). Food security was 60.6% in 2017, which improved to 83.3% and 89.0% in 2018 and 2019, respectively. Most households had (89.2%–91.2%) access to an improved toilet, around half of the adolescents (48.6%–51.6%) belonged to socially disadvantaged caste/ethnicity and around one‐third resided in the *terai*.

**Table 1:** Selected characteristics of unmarried adolescents and young women (10–22 years) in 2017, 2018 and 2019.

		2017		2018		2019	
		(*N* = 770)		(*N* = 682)		(*N* = 618)	
Characteristics	Categories	%	*N*	%	*N*	%	*N*
Potential associated factors							
Age median (Q1, Q3)		13.6	770	14.3	682	15.1	618
		(11.7, 15.6)		(12.5, 16.3)		(13.5, 17.1)	
Age category	10–14 years	66.4%	511	58.2%	397	48.4%	299
	15–19 years	33.6%	259	38.4%	262	46.4%	287
	≥ 20 years	0.0%	0	3.4%	23	5.2%	32
Education grade		6.0 (5.0, 8.0)	770	7.0 (6.0, 9.0)	682	8.0 (6.0, 9.0)	618
Median (Q1, Q3)							
Menarche status	Post‐menarche	51.9%	400	65.2%	445	78.3%	484
Lacto‐vegetarian	Yes	4.8%	37	5.3%	36	5.7%	35
IFA taken in the last 13 weeks	Yes	1.7%	13	16.9%	115	10.4%	64
Deworming tablets taken in the last 13 weeks^a^	Yes	—	—	30.9%	211	21.4%	132
Access to improved toilet	Yes	89.2%	687	91.2%	622	89.8%	555
Food insecurity	Food secure	60.6%	467	83.3%	568	89.0%	550
	Mildly insecure	19.6%	151	9.7%	66	6.0%	37
	Moderately insecure	16.9%	130	6.0%	41	4.5%	28
	Severely insecure	2.7%	21	1.0%	7	0.5%	3
Socioeconomic status	Lower	34.3%	264	35.0%	239	34.0%	210
	Middle	31.3%	241	32.0%	218	32.5%	201
	Higher	34.2%	263	33.0%	225	33.5%	207
Caste/Ethnicity	Socially disadvantaged	48.6%	374	51.6%	352	51.3%	317
	Brahmin/Chhetri	39.4%	303	38.6%	263	37.7%	233
	Others	12.1%	93	9.8%	67	11.0%	68
Agroecological zone	Mountain	16.0%	123	15.7%	107	14.6%	90
	Hill	51.2%	394	49.7%	339	50.5%	312
	Terai	32.9%	253	34.6%	236	35.0%	216
Nutritional status							
Dietary diversity mean (SD)		4.1 (1.2)	770	4.2 (1.2)	682	4.4 (1.2)	618
Minimum dietary diversity% (≥ 5 food groups)	Met	35.7%	275	40.2%	274	44.0%	272
Haemoglobin median (Q1, Q3)		12.8	758	12.7	681	12.8	616
		(12.0, 13.5)		(11.9, 13.5)		(12.0, 13.6)	
Anaemia %	Present	20.6%	156	25.1%	171	24.8%	153

Abbreviation: SD, standard deviation.

^a^ Not collected in 2017.

**Figure 1:**
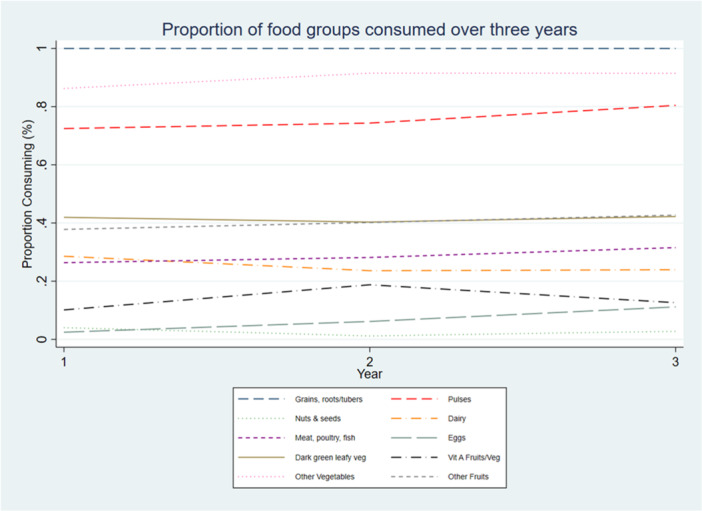
Consumption of 10 food groups in the last 24 h among 10–19 years in 2017, 10–21 years in 2018 and 10–22 years in 2019.

Mean DDS increased slightly over 3 years from 4.1 food groups in 2017 to 4.2 in 2018 and 4.4 in 2019. The proportion meeting the minimum dietary diversity of at least five food groups was 35.7% in 2017 increasing to 40.2% in 2018 and 44% in 2019. Diets consisted of grains, starchy roots/tubers, consistently consumed by the majority, followed by other vegetables and pulses. The least consumed were eggs and vitamin A‐rich fruits and vegetables (Figure 1). There was small to modest improvement in the proportion consuming other vegetables (86.2% in 2017, 91.5% at 2018 and 91.4% in 2019), pulses (72.5% in 2017, 74.3% at 2018 and 80.4% in 2019), while egg consumption, though low, showed an upward trend (2.5% in 2017, 6.2% at 2018 and 11.2% in 2019). Meat, poultry, and fish intake also increased slightly (26.4% in 2017, 28.2% at 2018 and 31.6% in 2019) but there was little to no improvement in the consumption of dark green leafy vegetables, other fruits, and nuts and seeds. Notably, dairy intake declined from 28.6% in 2017 to 23.6% in 2018 and 24.0% in 2019, while vitamin A rich fruits and vegetables consumption increased in 2018 (18.8%) from 10.1% but dropped in 2019 (12.6%) (Figure 1).

Mean haemoglobin was consistently 12.7–12.8 g/dL, slightly above the WHO anaemia threshold of 12.0 g/dL (World Health Organisation 2011). Anaemia prevalence was 20.6% in 2017, rising to 25.1% in 2018 and 24.8% in 2019.

### 3.1 Factors Associated With Dietary Diversity, Haemoglobin and Anaemia

Table 2 presents the unadjusted and adjusted regression results for DDS. In the adjusted dietary diversity model (*N* = 2068), adolescents living in *terai* (*β* = −0.95, 95% CI −1.14, −0.75) and hill regions (*β* = −0.63, 95% CI −0.79, −0.47) had substantially lower DDS than those living in the mountains. Adolescents from other castes (*β* = −0.40, 95% CI −0.59, −0.21) compared to Brahmin/Chhetri adolescents and those following a lacto‐vegetarian (*β* = −0.30, 95% CI −0.54, −0.07) versus a non‐vegetarian diet also had lower DDS. Compared to adolescents from food secure households, those from mildly, moderately and severely food insecure households consumed on average −0.23 (95% CI −0.38, −0.08), −0.34 (95% CI −0.51, −0.17) and −0.44 (95% CI −0.84, −0.04) respectively fewer food groups. Each unit increase in education (*β* = 0.07, 95% CI 0.03–0.10) was positively associated with the number of food groups consumed by adolescents. SES was not associated with DDS in the adjusted model (Table 2).

**Table 2:** Unadjusted and adjusted associations of potential factors with dietary diversity among adolescents and young women 10–22 years.

Variables	Categories	Dietary diversity				
		Unadjusted coefficient (95% CI)	*p*‐value	*N*	Adjusted coefficient (95% CI) (*N* = 2068)	*p*‐value
Age		0.04 (0.017–0.064)	< 0.001	2070	−0.02 (−0.05, 0.01)	0.225
Education		0.08 (0.05–0.10)	< 0.001	2070	0.07 (0.03–0.10)	< 0.001
Lacto‐vegetarian	No	Ref.		2070	Ref.	
	Yes	−0.34 (−0.60, −0.09)	0.008		−0.30 (−0.54, −0.07)	0.012
Food insecurity	Secure	Ref.		2069	Ref.	
	Mild	−0.16 (−0.31, −0.002)	0.047		−0.23 (−0.38, −0.08)	0.003
	Moderate	−0.29 (−0.47, −0.12)	< 0.001		−0.34 (−0.51, −0.17)	< 0.001
	Severe	−0.45 (−0.86, −0.03)	0.036		−0.44 (−0.84, −0.04)	0.032
Socioeconomic status	Lower	Ref.		2068	Ref.	
	Middle	−0.03 (−0.15, 0.10)	0.691		0.04 (−0.09, 0.17)	0.586
	Higher	−0.16 (−0.29, −0.03)	0.015		0.13 (−0.03, 0.28)	0.121
Caste/Ethnicity	Brahmin/Chhetri	Ref.		2070	Ref.	
	Socially disadvantaged	−0.22 (−0.34, −0.11)	< 0.001		−0.08 (−0.20,0.04)	0.199
	Other	−0.56 (−0.74, −0.38)	< 0.001		−0.40 (−0.59, −0.21)	< 0.001
Agroecological zone	Mountain	Ref.		2070	Ref.	
	Hill	−0.57 (−0.72, −0.42)	< 0.001		−0.63 (−0.79, −0.47)	< 0.001
	Terai	−0.90 (−1.06, −0.74)	< 0.001		−0.95 (−1.14, −0.75)	< 0.001

*Note:* These variables were assessed in unadjusted models but not retained in final models: occupation, owning a mobile phone, and participation in any clubs, gender of the household head, participant avoids certain foods during menstruation, urban/rural residency and data collection months.

Abbreviation: CI, confidence interval.

Table 3 shows results of the unadjusted and adjusted regression analysis for haemoglobin and anaemia. Living in the *terai* (*β* = −1.09 g/dL, 95% CI −1.32, −0.86) and hills (*β* = −0.23 g/dL, 95% CI −0.42, −0.03), following a lacto‐vegetarian diet (*β* = −0.33 g/dL, 95% CI −0.59, −0.08), being post‐menarche (*β* = −0.24 g/dL, 95% CI −0.37, −0.11) and belonging to a socially disadvantaged caste/ethnicity (*β* = −0.15 g/dl, 95% CI −0.29, −0.01) were associated with lower haemoglobin (Table 3). There was no association between access to improved toilet (*β* = 0.17 g/dL, 95% CI −0.01, 0.35) and haemoglobin in adjusted model. The bootstrap analysis, however, showed a positive significant association (*β* = 0.17 g/dL, 95% CI 0.02–0.32) (Supporting Information: Figure S1).

**Table 3:** Unadjusted and adjusted associations of potential factors with haemoglobin and anaemia among adolescents and young women 10–22 years.

Variables	Categories	Haemoglobin			Haemoglobin (*N* = 2053)		Anaemia (*N* = 2053)	
		Unadjusted coefficient (95% CI)	*p*‐value	*N*	Adjusted coefficient (95% CI)	*p*‐value	Adjusted odds ratio (95% CI)	*p*‐value
Age		−0.02 (−0.04, 0.01)	0.225	2055	0.01 (−0.02, 0.04)	0.434	1.07 (0.97–1.18)	0.178
Menarche status	Pre‐menarche	Ref.		2053	Ref.			
	Post‐menarche	−0.23 (−0.34, −0.12)	< 0.001		−0.24 (−0.37, −0.11)	< 0.001	2.26 (1.41–3.60)	0.001
Lacto‐vegetarian	No	Ref.		2055	Ref.			
	Yes	−0.42 (−0.68, −0.15)	0.002		−0.33 (−0.59, −0.08)	0.010	1.24 (0.56–2.75)	0.601
Access to improved toilet	No	Ref.		2054	Ref.			
	Yes	0.32 (0.13–0.50)	0.001		0.17 (−0.01, 0.35)	0.061	1.02 (0.57–1.81)	0.955
Socioeconomic status	Lower	Ref.		2053	Ref.		Ref.	
	Middle	−0.11 (−0.23, 0.01)	0.074		0.05 (−0.07, 0.17)	0.400	0.75 (0.48–1.17)	0.201
	Higher	−0.37 (−0.51, −0.22)	< 0.001		0.06 (−0.09, 0.22)	0.424	0.79 (0.46–1.33)	0.371
Caste/Ethnicity	Brahmin/Chhetri	Ref.		2053	Ref.		Ref.	
	Socially disadvantaged	−0.31 (−0.46, −0.16)	0.0001		−0.15 (−0.29, −0.01)	0.040	1.87 (1.19–2.95)	0.007
	Others	−0.30 (−0.52, −0.08)	0.0082		0.00 (−0.21, 0.22)	0.986	1.49 (0.76–2.91)	0.241
Agroecological zone	Mountain	Ref.		2055	Ref.		Ref.	
	Hill	−0.25 (−0.45, −0.05)	0.013		−0.23 (−0.42, −0.03)	0.025	1.72 (0.88–3.36)	0.115
	Terai	−1.13 (−1.34, −0.92)	< 0.001		−1.09 (−1.32, −0.86)	< 0.001	11.89 (5.50–25.70)	< 0.001

*Note:* Assessed in unadjusted models but not retained in final models: education, knowledge of water treatment methods, handwashing practice, DDS, minimum dietary diversity (>=5 food groups), consumption of green leafy vegetables, iron folic acid supplementation, anthelminthic deworming, household food insecurity, gender of the household head, urban/rural residence and data collection months.

Three of these factors—living in the *terai* region (AOR 11.89, 95% CI 5.50–25.70), being post‐menarche (AOR 2.26, 95% CI 1.41–3.60) and belonging to a socially disadvantaged caste/ethnicity group (AOR 1.87, 95% CI 1.19–2.95) were associated with higher odds of anaemia. Following a lacto‐vegetarian diet, and access to improved toilets were not significantly associated with anaemia (Table 3).

## 4 Discussion

Our analysis draws on data from a longitudinal panel study of adolescent girls and young women in Nepal to examine changes in and factors associated with dietary diversity, haemoglobin, and anaemia over 3 years (2017–2019). DDS remained consistently low, with diets dominated by starchy staples and limited consumption of micronutrient‐rich foods, consistent with findings from Nepal (Rai et al. 2023) and Pakistan (Baxter et al. 2022). This pattern suggests adolescents and young women have inadequate micronutrient intake to meet their nutritional needs (Das et al. 2017; Ty Beal et al. 2024; Arimond et al. 2010). Haemoglobin levels, similarly, remained between 12.7 g/dL and 12.8 g/dL, and anaemia emerged as a moderate public health concern in our sample (World Health Organisation 2025), with prevalence increasing from 20.6% to 24.8% as the girls aged from 13.6 to 15.1 years, on average, and entered menarche. These findings align with those of Ford et al. (2022), although our prevalence was lower than the national prevalence of 39% among those 15–19 years in 2022 (Ministry of Health Nepal, New ERA, and ICF 2023). The increase in anaemia prevalence may be due to more girls entering menarche, alongside low IFA coverage, and other factors. Common factors associated with both DDS and haemoglobin/anaemia were living in the *terai*, belonging to less advantaged caste/ethnic groups, and following a lacto‐vegetarian diet. Additionally, education and food security were key factors associated with DDS, while post‐menarche status was associated with haemoglobin/anaemia, and access to improved sanitation facilities was linked with haemoglobin levels.

Despite the *terai* having a larger area of arable land, higher agricultural production and better availability of diverse foods (Krupnik et al. 2021), *terai* participants had lower DDS. This may be because women and girls in the *terai* experience restricted mobility and agency (Morrison et al. 2018, 2023; Madjdian et al. 2022), strict gender‐based food allocation (Harris‐Fry et al. 2018), and tend to eat least and last (Morrison et al. 2021, 2023). Living in the *terai* was also associated with nearly 1 g/dL lower haemoglobin and almost 12 times higher odds of anaemia, calling for specific interventions for adolescents and young women in this region. Higher burden of infections and environmental toxins in the *terai* may lead to environmental enteric dysfunction, impairing nutrient absorption and iron stores (Mehata et al. 2022; Andrews‐Trevino et al. 2022; Smith et al. 2016; Regassa et al. 2023). We also found that access to an improved toilet was associated with higher haemoglobin levels, aligning with previous evidence that improved WASH facilities and practices reduces helminth infections, which can impair nutrient absorption and contribute to anaemia (Mehata et al. 2022; Strunz et al. 2014; Ngure et al. 2014). Despite these potential causal pathways, we found no association between deworming or IFA and haemoglobin/anaemia, likely due to low coverage in our population and thus limited power to detect a difference. Programmes targeting adolescents in the *terai* should address sociocultural barriers that limit access to diverse and nutrient‐rich foods, particularly the unequal intra‐household distribution of animal‐source foods (e.g., prioritising men) (Harris‐Fry et al. 2018), while also strengthening girls' nutritional agency, and education (Morrison et al. 2023; Hargreaves et al. 2022). Additionally, improving the availability and affordability of iron‐rich and fortified foods, increasing IFA and deworming coverage, and improving WASH infrastructure are critical strategies for reducing anaemia in this region (Chaparro and Suchdev 2019; Hargreaves et al. 2022; Rai et al. 2019).

Consistent with previous studies on dietary diversity (Rai et al. 2023; Sharma and Smieliauskas 2022; Bennett et al. 2008) and haemoglobin/anaemia from Nepal (Rai et al. 2023; Ford et al. 2022), we found adolescents and young women from less advantaged caste/ethnicity had lower DDS, lower haemoglobin levels, and a higher risk of anaemia, compared to advantaged peers. These disparities may stem from systemic barriers including generationally limited access to education, employment, healthcare and other essential resources, that contribute to broader social deprivations (Thapa et al. 2021; Ghimire 2019; Organization 2008). This is supported by evidence from India, where caste‐based economic constraints limited access to fruits and vegetables consumption (Choudhury et al. 2021), while studies in Nepal further highlight that social exclusion and disparities in education and healthcare exacerbate these limitations (Thapa et al. 2021; Mosse 2018; Madjdian and Bras 2016). Additionally, cultural dietary norms may restrict access to diverse, nutrient‐dense foods (Madjdian and Bras 2016). Recent research also suggests that upper caste women, compared to lower caste women, are more likely to adopt recommended nutrition practices (Cunningham et al. 2024). While improvements in health services, sanitation, education, and wealth have contributed to Nepal's success in maternal and child nutrition (Cunningham et al. 2017), similar progress is needed to address persistent caste and ethnic disparities in adolescent nutrition. Although there has been some research and documentation of development programmes focused on disadvantaged groups (Thapa et al. 2021; Ghimire 2019; Cunningham et al. 2024; Asian Development Bank 2020), the mechanisms linking caste/ethnicity with adverse nutritional outcomes remains underexplored. A deeper understanding of how caste‐ and ethnicity‐based exclusion limits nutritional opportunities is crucial in improving programme effectiveness.

Consuming a lacto‐vegetarian diet which excludes flesh foods (meat, poultry and fish) and eggs was associated with lower DDS in our study, as these two food groups are part of the MDD‐W indicator. However, it is important to note that only approximately 5% of girls reported following such a diet, so while this dietary pattern may contribute to low DDS, it affects only a small proportion of the adolescent girl population. This dietary pattern, often rooted in religious or cultural practices, may limit the intake of key micronutrients. A systematic review found that vegetarians and vegans tend to have lower blood/serum vitamin A, vitamin E, ferritin, selenium, iodine and zinc (Dawczynski et al. 2022). In line with this, we also found that adherence to a lacto‐vegetarian diet was associated with lower haemoglobin levels, likely due to insufficient intake of bioavailable iron, particularly haem iron (Chaparro and Suchdev 2019), as also observed in a study from Bangladesh linking meat consumption with higher haemoglobin levels (Ahmed et al. 2008). Addressing these nutritional gaps requires culturally sensitive strategies that promote dietary diversity and iron‐rich sources within vegetarian diets, including the use of locally available pulses, vegetables, and grains. Interventions should also incorporate education on enhancing iron absorption such as pairing non‐haem iron sources with vitamin C‐rich foods and reducing intake of absorption inhibitors like tea, coffee, and high‐phytate foods (Saville et al. 2023). Supporting the nutritional adequacy of vegetarian diets can also contribute to sustainable food systems and climate change mitigation efforts (Hargreaves et al. 2022).

Other factors specifically associated with DDS were years of education and food insecurity. In line with our finding that higher educational achievement was associated with higher DDS, a study from Nepal found that more educated adolescents were more likely to meet the recommended intake of fruits and vegetables (Singh et al. 2019). Education may improve diets by enhancing literacy, increasing health and nutrition knowledge (Wachs 2008). It may also contribute to improved social status, greater employment opportunities and enhanced agency (Morrison et al. 2023; Diamond‐Smith et al. 2022; Gupta 1995), all of which support access to healthier diets. However, education alone may not be sufficient if prevailing social norms continue to limit girls' access to diverse and nutrient‐rich foods. Social and behaviour change interventions are needed to shift perceptions of adolescents' value within households and improve gendered food allocation (Morrison et al. 2023). Additionally, we found that DDS decreased with increasing food insecurity, consistent with studies in Nepal (Diamond‐Smith et al. 2020; Diamond‐Smith et al. 2022; Madjdian and Bras 2016). While improving household food security could support diverse diets, programmes must be adolescent‐centred, recognising that intra‐household food allocation may still disproportionately favour men (Morrison et al. 2023), or women in higher household positions such as mothers‐in‐law, over adolescent girls (Harris‐Fry et al. 2018; Madjdian and Bras 2016).

Haemoglobin and anaemia outcomes were specifically associated with menarche status. Post‐menarche adolescent girls had lower haemoglobin and higher odds of anaemia compared to pre‐menarche girls, consistent with a review of anaemia risk factors (Wiafe et al. 2023). This may reflect increased physiological demands for iron during menstruation (Das et al. 2017; Ty Beal et al. 2024), compounded by dietary deficiencies in iron and other nutrients essential for red blood cell production (Chaparro and Suchdev 2019). Diets in our sample mainly included starchy staples and pulses with low intakes of vitamin‐A‐rich vegetables, fruits, and animal‐source foods. To address these gaps, interventions should include micronutrient and/or fortified food supplementation, alongside behaviour change strategies to promote healthier dietary practice focusing on building iron stores pre‐menarche and replenishing iron post‐menarche, ensuring that all adolescent girls are supported to maintain adequate iron status and reduce anaemia risk (Ahmed et al. 2008).

### 4.1 Strengths and Limitations

Our study is the first to examine Nepalese adolescent girls' diets, haemoglobin levels, and anaemia longitudinally over 3 years. However, our study has some limitations. Our *terai* region findings show wide confidence intervals for haemoglobin/anaemia, indicating high variability and uncertainty. The difference in CI between the final and bootstrap models for improved toilet and haemoglobin may result from potential outliers and should therefore be interpreted with caution. Due to the lack of an adolescent‐specific instrument, dietary diversity was assessed using the WHO/FAO tool for women aged 15–49, and there may be some recall bias associated. There may be residual confounding due to inaccurate dietary diversity measure and unmeasured variables. Haemoglobin was measured using capillary blood, which may differ from venous samples. Haemoglobin and anaemia were adjusted for smoking and elevation based on the WHO 2011 guidelines (World Health Organisation 2011) as the data were collected before the 2024 guidelines were endorsed. Analyses using the updated 2024 guidelines, however, might yield different results (Casal et al. 2024). We measured anaemia only as a cross‐sectional status at each visit, so we could not distinguish prevalent from incident cases. In future re‐analysis of these data, girls who are non‐anaemic at baseline could be followed until first anaemia diagnosis for an incidence estimate, and insight into when anaemia develops. Our findings may have been confounded by exposure to nutrition interventions such as the described pilot adolescent nutrition programme for adolescents or community‐wide programmes, such as radio campaigns. While loss to follow‐up was observed in the sub‐sample, it did not differ by dietary diversity, suggesting minimal bias in exposure assessment. However, participants lost in 2018 had higher haemoglobin levels at that time point, which may have introduced some bias in anaemia estimates during later follow‐ups.

## 5 Conclusion

This study showed that adolescent girls in Nepal's *terai* and hill region and from disadvantaged caste/ethnicity groups face particularly low dietary diversity, persistently low haemoglobin levels, and those in *terai* and from disadvantaged caste/ethnicity groups face an increasing rate of anaemia over time. We also found that factors such as education, household food security were linked to better diets and access to improved sanitation to haemoglobin levels. Girls who had reached menarche had lower haemoglobin and a higher risk of anaemia, highlighting a critical window for intervention. Our findings highlight the urgent need for coordinated, multisectoral policy action and increased investment in adolescent health. Government stakeholders and development agencies should prioritise adolescent nutrition within national health, education, and social protection frameworks. Potential effective strategies include scaling up and strengthening culturally appropriate food and micronutrient supplementation programmes, school‐based nutrition education, and access to sanitation facilities in homes, schools and health facilities. Special attention should be given to delivering interventions around menarche and continuing throughout adolescence, ensuring programmes effectively reach disadvantaged caste/ethnic groups, by reducing barriers to access, and prioritising high burden *terai* and hill regions to address low dietary diversity, post‐menarche decline in haemoglobin levels and increase in anaemia.

Further research is critical to inform policy and programmes. Studies examining how education and food insecurity interact with social norms and intra‐household food dynamics in this population can further clarify how to improve dietary diversity and adolescent nutrition. Research investigating disparities across caste/ethnicity and agroecological zones is needed to identify context‐specific challenges and opportunities. Mixed‐method studies can also help unpack the socio‐cultural, infection‐related, and WASH‐related pathways contributing to poor nutritional outcomes in *terai*, as well as systemic barriers in access to resources and opportunities among disadvantaged groups. Addressing these persistent nutritional challenges through robust research and responsive policy action is essential to improve adolescent wellbeing and support national development goals.

## Author Contributions

Anjana Rai conceptualised the study, and all authors contributed to the study conception and design. Data collections were performed by Kenda Cunningham and Ramesh P. Adhikari through *Suaahara II*, and analyses were performed by Anjana Rai. The first draft of the manuscript was written by Anjana Rai and all authors contributed to revisions of the manuscript. All authors read and approved the final manuscript.

## Conflicts of Interest

The authors declare no conflicts of interest.

## Supporting information


**Figure S1:** Regression results for haemoglobin (*n* = 2053) and anaemia (*n* = 2053).

## Data Availability

The data used in this study were obtained from the Suaahara‐II project of Helen Keller International. Access to the data may be possible upon reasonable request and with permission from Helen Keller International. Interested parties should contact them directly or reach out to the corresponding author.
