# Supplementary figures and images for: Dietary Diversity, Haemoglobin and Anaemia in Nepali Adolescent Girls: A Longitudinal Study
Source: Matern Child Nutr. 2025 Aug 29;22(1):e70090. doi: 10.1111/mcn.70090 (PMC12893519; doi:10.1111/mcn.70090)

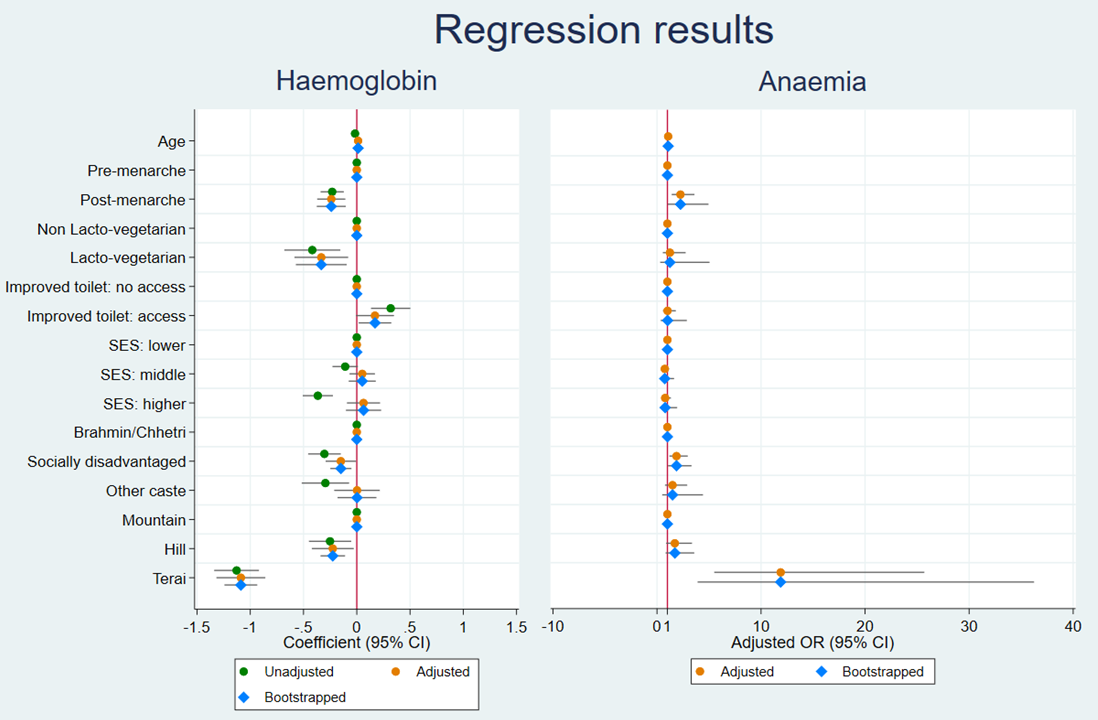

Supplement: Supplementary file 1 — Figure S1: Regression results for haemoglobin (n = 2053) and anaemia (n = 2053). [file MCN-22-e70090-s001.png]
